# High birth weight and perinatal mortality among siblings: A register based study in Norway, 1967-2011

**DOI:** 10.1371/journal.pone.0172891

**Published:** 2017-02-28

**Authors:** Petter Kristensen, Katherine M. Keyes, Ezra Susser, Karina Corbett, Ingrid Sivesind Mehlum, Lorentz M. Irgens

**Affiliations:** 1 Department of Occupational Medicine and Epidemiology, National Institute of Occupational Health, Oslo, Norway; 2 Department of Community Medicine, Institute of Health and Society, University of Oslo, Oslo, Norway; 3 Department of Epidemiology, Mailman School of Public Health, Columbia University, New York, NY, United States of America; 4 New York State Psychiatric Institute, New York, NY, United States of America; 5 Department of Global Public Health and Primary Care, University of Bergen, Bergen, Norway; 6 Medical Birth Registry of Norway, Norwegian Institute of Public Health, Bergen, Norway; Centre Hospitalier Universitaire Vaudois, FRANCE

## Abstract

**Background:**

Perinatal mortality according to birth weight has an inverse J-pattern. Our aim was to estimate the influence of familial factors on this pattern, applying a cohort sibling design. We focused on excess mortality among macrosomic infants (>2 SD above the mean) and hypothesized that the birth weight-mortality association could be explained by confounding shared family factors. We also estimated how the participant’s deviation from mean sibling birth weight influenced the association.

**Methods and findings:**

We included 1 925 929 singletons, born term or post-term to mothers with more than one delivery 1967–2011 registered in the Medical Birth Registry of Norway. We examined z-score birth weight and perinatal mortality in random-effects and sibling fixed-effects logistic regression models including measured confounders (e.g. maternal diabetes) as well as unmeasured shared family confounders (through fixed effects models). Birth weight-specific mortality showed an inverse J-pattern, being lowest (2.0 per 1000) at reference weight (z-score +1 to +2) and increasing for higher weights. Mortality in the highest weight category was 15-fold higher than reference. This pattern changed little in multivariable models. Deviance from mean sibling birth weight modified the mortality pattern across the birth weight spectrum: small and medium-sized infants had increased mortality when being smaller than their siblings, and large-sized infants had an increased risk when outweighing their siblings. Maternal diabetes and birth weight acted in a synergistic fashion with mortality among macrosomic infants in diabetic pregnancies in excess of what would be expected for additive effects.

**Conclusions:**

The inverse J-pattern between birth weight and mortality is not explained by measured confounders or unmeasured shared family factors. Infants are at particularly high mortality risk when their birth weight deviates substantially from their siblings. Sensitivity analysis suggests that characteristics related to maternal diabetes could be important in explaining the increased mortality among macrosomic infants.

## Introduction

Birth weight is a strong predictor of stillbirth and neonatal mortality. The relation between birth weight and mortality is an inverted J-pattern with a steep mortality slope among those small at birth (the long arm), and a shorter slope among high birth weight infants (the hook) [1,2]. Since the first documentation of this pattern [3], substantial effort in different scientific disciplines has been posited in order to provide explanations, including evolutionary biology [1–3], epidemiology [4–8], clinical sciences [9–20], animal sciences [21], and sociology [22]. Suggestions across these disciplines include both causal and noncausal explanations. A causal explanation could be that there is an optimum population target weight with deviations being associated with higher mortality levels, or that characteristics at delivery might mediate a mortality risk [1,10]. A non-causal effect could be due to the influence of rare yet extremely strong confounding factors, or to more common interacting factors that are determinants of size and survival [6–8]. To date, non-causal pathways have not fully explained the association, yet firm consensus and mechanisms for causal effects remain elusive. While the relation between low birth weight and mortality has been extensively documented, macrosomia is receiving renewed attention because the prevalence is increasing in most countries, and because of its association with perinatal death and other short-term and long-term adverse health outcomes [10]. Infants in pregnancies with maternal hyperglycemia, pregestational and gestational diabetes mellitus, obesity, and metabolic syndrome are more likely to be macrosomic at birth [15–20], and maternal diabetes or obesity are associated with perinatal death [16–19]. Several [11,12,14,23,24] studies report elevated stillbirth or neonatal mortality rates in association with macrosomia. There are several mechanisms through which macrosomia may plausibly increase mortality, including epigenetic processes and placental function [25], maternal hyperglycemia [15], as well as labor and delivery complications and birth injury due to large body size [12–14,23].

Epidemiological [4–8] and biological [9–14,23,24] approaches aimed at explaining the relation between birth weight and perinatal mortality have mainly been limited to maternal or offspring factors during the actual pregnancy. Commentaries have suggested looking beyond this, searching for contextual conditions in the family that could illuminate the relationship between size and survival [26]. Indeed, as early as 1951, within-family clustering of birth weight and mortality was shown [27]. Since then, familial birth weight clustering has been demonstrated between siblings [28–32], mother and child [32,33], and first cousins [31]. Size at birth in one family member and early-life mortality in relatives can also cluster [34–36].

Using family data to separate individual and family effects of birth weight on perinatal outcomes is a way to potentially illuminate some of these explanations [37,38]. This approach has been used in several studies, showing that sibling [39–43] or maternal [44] birth weight influences the association between infant birth weight and mortality. These studies [39–44] suggest that infants are at increased risk for mortality if their birth weight is lower than that of their siblings or parents at birth. This finding is in agreement with a biologic interaction between factors determining birth weight in the individual and factors determining the optimum birth weight in the family. Two gaps in knowledge remain, however. First, with the exception of twin studies [45], conditioning on shared but unmeasured family confounders has been incomplete, which is necessary to rule out unmeasured confounding. Such control can be achieved in sibling discordance studies [37]. Second, the influence of deviations from mean family birth weight on the relation between macrosomia and mortality (the hook) has barely been addressed in prior studies [39–44]. We based the present study on births in the Medical Birth Registry of Norway (MBRN). Our objective was to examine the relationship between birth weight and perinatal mortality (PNM), taking a number of covariates into consideration. We intended to study the entire birth weight distribution, with a particular emphasis on macrosomia and the hook of the inverted J. The a priori hypothesis was that the higher PNM in the macrosomic segment was confounded by unmeasured, shared family factors, as found earlier for macrosomia in association with lower intelligence [46]. We also wanted to estimate how the participant’s deviation from mean sibling birth weight influenced the birth weight-mortality association. This influence was quantified by estimating the population attributable fraction (PAF) of PNM in association with deviance from mean sibling birth weight.

## Materials and methods

### Study population

Since 1967, the MBRN has recorded data on all births in the country [47]. The national identification number assigned to all residents (mothers, fathers, infants) enables the establishment of family files with linkage of birth record data. This allows the estimation of combined effects of family members’ birth weights. The data include all 2 645 886 births notified in the MBRN between 1967 and 2011. We aimed at examining maternal families with at least two infants with available data on exposure (birth weight) and outcome (perinatal death), where at least one infant (participant) should be singleton and born at term or post-term (later than 36 completed gestational weeks).

**Fig 1:**
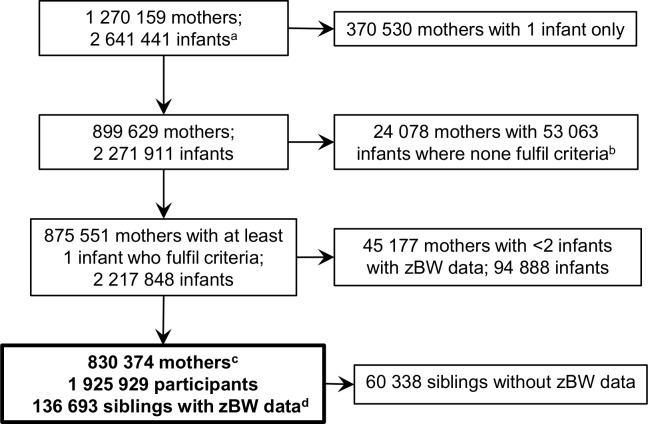
Study population. zBW: z-score birth weight, standardized for year of birth, sex, birth order, gestational week, and plurality. ^a^ Not including 4445 infants with unidentified mothers. ^b^ None of mother’s infants fulfilled the criterion of being singleton born at term or post-term, and having zBW data, because they were preterm, plural births, missing gestational age, missing birth weight, or combinations of the above. ^c^ Mothers who gave birth to a total of 2 122 960 infants (range 2–16, mean 2.56). ^d^ Siblings who did not fulfil participation criteria because they were preterm (68%), plural born (19%), or both (13%), but who contributed with zBW data.

Details of the establishment of the study population are provided in Fig 1. Births to unidentified mothers and births to mothers who did not give birth to participant(s) and at least two infants with birth weight data were excluded; 830 374 mothers with 2 122 960 infants (range 2–16) remained. Infants who fulfilled the singleton and gestational age criteria (N = 1 925 929) constituted the study participants. In addition, the participants had 136 693 preterm or plural born siblings who contributed with family-level data. The rationale for not including preterm infants as participants was that they would probably include some with a four-week error in gestational week due to bleeding early in pregnancy [48]. This could result in a contamination of the sparsely populated strata of the most extreme macrosomic categories by infants who truly were normal weight and term born. Plural births were not included because it would complicate performance and assessment of the analyses.

The Regional Committee for Medical Research Ethics approved the study.

### Variables

#### Outcome and main exposure variable

The study outcome was perinatal death, defined as stillbirth (death before or during delivery), or early neonatal death (death within one week after delivery) [49]. Additional analyses were run with either stillbirth or early neonatal death as outcome. Perinatal deaths per 1000 births (PNM) constituted the group-level mortality measure.

The main exposure variable was birth weight. In order to compare all participants and siblings, a z-score birth weight was standardized for sex, parity, gestational week, year of birth, and plurality, into a zBW variable with mean = 0 and standard deviation (SD) = 1. We categorized zBW into 10 ordered levels, as well as a broader categorization of infants into microsomic (zBW <–2), normosomic (zBW –2 to +2), and macrosomic (zBW >+2).

#### Covariates based on family characteristics

We constructed four variables based on the mothers’ births: “mean sibship zBW”, “deviance from mean sibling zBW”, “paternity”, and “sibship size”. We defined mean sibship zBW as the mean zBW of all the mother’s infants, categorized into four levels. Deviation from mean sibling zBW was defined as the difference between a participant’s zBW and mean zBW of all other infants to the mother. We ordered this variable into five categories: ≥1 SD lower than sibling mean zBW; 0.5 to 1 SD lower; <0.5 SD difference; 0.5 to 1 SD higher; and ≥1 SD higher. The middle three categories were collapsed into a “<1 SD difference” category in some analyses. The paternity variable was based upon the father’s identity for infants in the maternal family. The main purpose was to assess if the paternal contribution to birth weight in the sibship came from one or more fathers. We applied three categories: “same father” if all infants with zBW data in the maternal families had the same father, “not same father” if some infants with zBW data had different fathers, and “uncertain” in the 1500 (0.2%) maternal families with a combination of the same father and unidentified fathers. Sibship size was a count of all infants to the mother.

#### Other covariates

We considered a number of other covariates based on their potential to influence the zBW-PNM association [10–14], such as year of birth, sex, birth order, gestational weeks, birth defect, preeclampsia, and maternal characteristics (age, marital status, diabetes mellitus, chronic disease). Gestational week was based on date of last menstrual period until 1999 and mainly on ultrasound measurements thereafter. Birth defects were categorized as ICD-8 codes 740–759 (1967–1998) and ICD-10 Q codes (1999–2011) [50,51]. Maternal chronic disease was a dichotomous variable registered in the birth record. Data on maternal smoking, placental weight, and maternal prepregnancy body mass index (BMI) were only available for recent years. In 1999, MBRN introduced a new birth notification form with improved data quality for maternal diabetes mellitus and other items, as well as new variables such as maternal smoking and placental weight [47]; analyses using these variables were restricted to 1999–2011 births. The dichotomous maternal diabetes variable included both pregestational and gestational diabetes. We divided maternal smoking into never smokers, former smokers, current smokers, and non-responders. We also computed a birth/placenta weight ratio variable divided into quartiles. BMI data, which we ordered into four levels, were available in a 40% sample of maternity units from 2006 onwards and analyses using this variable was restricted to this subset.

Factors considered to have a potential of mediating a causal zBW effect [12–14,23] were obstetrical procedure, dystocia, and birth injury.

### Causal diagram

**Fig 2:**
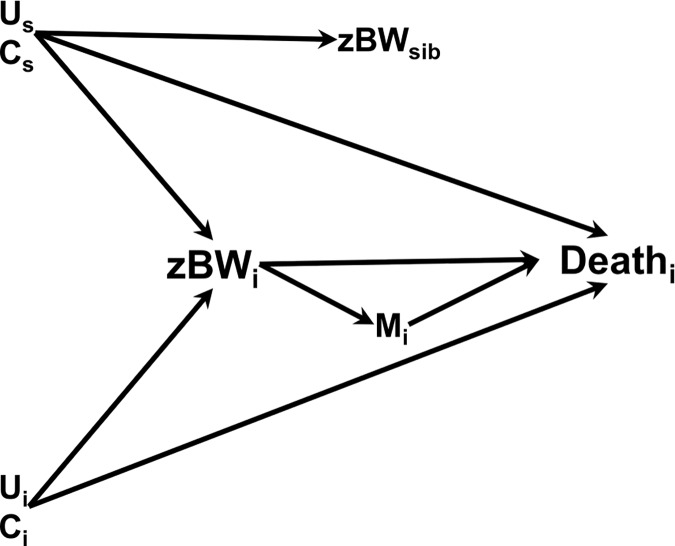
Causal diagram illustrating the relation between perinatal death and birth weight. zBW: z-score birth weight, standardized for year of birth, sex, birth order, gestational week, and plurality. Subscripts: _i_ relates to index infant (participant); _s_ relates to shared family factors; _sib_ relates to sibling(s).U: Unmeasured confounders. U_s_ are shared factors in the family, e.g., genetic factors, stable maternal health or metabolic characteristics, stable socioeconomic characteristics. U_i_ are individual factors, e.g., placental function, pregnancy-specific maternal health or metabolic characteristics, socioeconomic characteristics changing across pregnancies.C: Measured confounders. C_s_: mean sibship zBW (zBW of all mother’s births), paternity, number of infants in sibship; C_i_: birth defect, preeclampsia, maternal age, maternal marital status, maternal diabetes, and maternal chronic disease.M_i_: Individual mediating factors (e.g., instrumental delivery procedures, prolonged labor, or birth injuries) whereby zBW_i_ could cause perinatal death among infants who were alive at start of the birth.

In order to explain our analytic choices, we made a causal diagram where the roles of the covariates with respect to the relation between zBW and PNM were depicted (Fig 2). Here, the roles of measured confounders C and unmeasured confounders U are included. We assumed no interaction between the potential confounders, as is common in causal analysis. M_i_ are mediating factors by which birth weight could cause death during delivery. This mediating pathway could only apply to stillbirths during delivery and early neonatal deaths. For stillbirths before delivery, the same factors could be common effects (colliders) of birth weight and death [52]. As an example, the combination of fetal death and macrosomia could be an indication for birth induction. Viewing cesarean section as a mediating factor that could explain a mechanism between macrosomia and death would be entirely wrong, which could create selection bias [52] rather than explaining a causal effect.

### Data analysis

The main analytical aim was to estimate individual-level associations between perinatal death and zBW, with emphasis on macrosomia, taking sibling zBW into account.

We used Stata/SE 13.1 software (Stata Corporation, College Station, Texas). The zBW variable was created by z-transforming and standardizing birth weight for sex, parity (5 categories), gestational week, year of birth, and plurality (2 categories) using Stata’s *rowsort* command. Descriptive characteristics of zBW, the covariates, and their relations to birth weight and perinatal death were calculated in ordinary tabular analysis.

#### Associations between perinatal death and zBW

In the analyses of individual-level associations between death and zBW, we applied two analytical procedures. Both included mother’s identity as the grouping variable. First, we applied random intercept panel-data logistic regression in the entire study population or in selected population subgroups. Second, we implemented a conditional (fixed-effects) logistic regression in the 7686 families (23 546 infants) with discordant perinatal survival experiences. The fixed-effects approach is essentially a conditional analysis estimating differences within sibships, and, contrary to the random-effects models that only control for factors included in the model, all shared (family) factors are invariant and controlled for by design. The advantage of fixed-effects is balanced by lower statistical power. Both procedures yielded risk coefficients (ln OR) and 95% confidence intervals (CI). Because we had emphasis on macrosomia, we ran analyses both with 10-level zBW and zBW where the three macrosomic zBW levels >+2 were collapsed. The zBW category (+1 to +2) with the lowest crude PNM served as reference.

We fit three random-effects models and one fixed-effects model. Details of the four models are outlined in Table 1. Model 1 was a crude analysis including categorical zBW only. Measured covariates, shared or individual, considered by us to be potential confounders, with the exception of factors already included in the standardization of birth weight, were added in Model 2. Model 2 aimed at separating individual and family zBW effects by adding mean sibship zBW. This is according to Begg and Parides’ model 2 [38], except that we categorized the variable due to its non-linear pattern with PNM. Because data on maternal smoking, placenta weight and maternal BMI were restricted to recent years, we ran additional analyses with these potential confounders in subset populations. In Model 3, three mediators were added to the factors in Model 2 in an analysis with early neonatal death as outcome. The small fraction of infants who experienced stillbirth were not at risk and were excluded in Model 3. The influence of mediation was assessed applying the difference method [53] by comparing zBW coefficients in Model 3 and a model without the mediators. Model 4 was a fixed-effects analysis including the same variables as in Model 2, except that shared factors, measured or unmeasured, were invariant by design.

**Table 1:** Characteristics of four models applied in the analyses.

Characteristics	Model 1	Model 2	Model 3	Model 4
Population	1 925 929 infants in 830 374 sibships	1 925 929 infants in 830 374 sibships	1 920 254 live born infants in 830 124 sibships	23 546 infants in 7686 sibships
Exposure^a^	zBW, 10 categories; macrosomia	zBW, 10 categories; macrosomia	Macrosomia	zBW, 10 categories
Outcome	Perinatal death	Perinatal death; stillbirth; early neonatal death	Early neonatal death	Perinatal death
Grouping variable	Mother’s identity	Mother’s identity	Mother’s identity	Mother’s identity
Potential confounders, shared^a^^,^^b^	Not included	Included	Included	Invariant
Potential confounders, individual^a^^,^^c^	Not included	Included	Included	Included
Mediators^a^^,^^d^	Not included	Not included	Included	Not included
Analysis method	Panel data random-effects logistic regression	Panel data random-effects logistic regression	Panel data random-effects logistic regression	Conditional (fixed-effects) logistic regression

zBW: z-score birth weight, standardized for year of birth, sex, birth order, gestational week, and plurality.

^a^ Variable details provided in Table 2.

^b^ Mean sibship zBW (zBW of all mother’s births; four levels), paternity, sibship size (mother’s number of births).

^c^ Birth defect, preeclampsia, maternal age, maternal marital status, maternal diabetes, maternal chronic disease.

^d^ Obstetrical procedure, dystocia, birth injury.

In this way, we were able to control for measured individual confounders and for measured and unmeasured shared confounders. This left the role of unmeasured individual confounders unexplained. The role of mediators was assessed for early neonatal death only.

We conducted analyses both in the total population and in subgroups. The most important was a comparison of early (1967–1998) and more recent (1999–2011) births, because aforementioned potential confounders were only measured in the more recent years.

The initial analyses revealed that associations between perinatal death and macrosomic categories were considerably stronger in pregnancies recognized as diabetic compared to non-diabetic pregnancies. We investigated this in a random-effects analysis with an interaction term of diabetes and zBW categories.

Differences in associations in mutually exclusive subgroups were tested using the approach of Altman and Bland [54]. We considered differences with two-sided p-values <0.05 to be statistically significant.

#### The influence of deviation from mean sibling zBW on the association between perinatal death and zBW

To obtain estimates of the modifying strength of sibling zBW on the zBW-PNM association, we computed a 30-level interaction variable combining the 10-level zBW variable and the three-level deviance from mean sibling zBW variable. We substituted this interaction variable for the zBW and the mean sibship zBW variable in models where the potential confounders in Model 2 were included. Our choice of reference was zBW +1 to +2 and <1 SD different from sibling mean zBW. This analysis was mainly done to examine if deviance from mean sibling zBW influenced mortality risk among macrosomic infants, but also to confirm studies that have shown that small and normal-sized infants have an added risk when they are of smaller size than their siblings [39–43].

The impact of sibling zBW on the zBW-PNM relation in the total population and selected subgroups was assessed by estimating PAFs. This was done in unconditional logistic regression models that included the zBW categories, the 5-level deviance from mean sibling zBW variable, and the potential confounders from Model 2. Using Stata’s *punaf* procedure, we were able to compare the observed population PNM_O_ with the hypothetical PNM_H_ that would have been experienced if all had had unchanged zBW category but the same PNM risk as that of groups with similar (<0.5 SD different) zBW as their siblings. The *punaf* procedure yielded PAF estimates = 1 –PNM_H_/PNM_O_ with 95% CI [55].

#### Sensitivity analysis

Three issues were considered in sensitivity analyses.

First, we assumed that the deviance from mean sibling zBW variable was a proxy for the individual zBW difference from an unknown programmed optimum birth weight. Accordingly, we did not differentiate between elder and younger siblings (Fig 2). However, if the effect was a consequence of mechanisms triggered during the mother’s earlier pregnancies rather than a stable, programmed family characteristic, our approach could distort the results. This was assessed by comparing the modifying role of sibling zBW on the zBW-PNM relation separately for the mothers’ first born and last born infant. The former group would only have siblings born after the participant and the latter group would only have siblings born before the participant.

Second, we assumed that the difference from mean sibship zBW variable was an indicator of deviance of own birth weight from an unknown programmed optimum birth weight, but only an imperfect one. We expected that this indicator would be better for the 726 584 families with the same mother and father than for the whole population, because of the father’s genetic contribution to offspring birth weight. We also assumed that deviance from mean sibling zBW would be a better indicator for large (>2 siblings) than for small (1–2 siblings) families. These indicator quality analyses were performed by comparing PAFs in the subsets, assuming larger impact for full sibling families and large families.

Third, we assumed that conditions related to maternal diabetes and metabolic disturbances were underreported in the MBRN, furthermore, that a similar interaction could be expected for such unrecognized conditions as for the observed diabetes variable. We investigated the impact of such underreporting by creating a hypothetical diabetes variable with higher prevalence than the observed one. This was achieved by changing status from non-diabetic to hypothetical diabetes for chosen fractions of the population. This procedure was done randomly within each specific zBW-PNM stratum, securing that zBW distribution and zBW-specific PNM were kept similar to the observed diabetes variable. The random-effects associations were then compared for observed diabetes and the more prevalent hypothetical diabetes variables.

## Results

Table 2 outlines the distribution of the exposure variable and covariates, including details of their definitions and categorizations. Grand mean birth weight in the study population was 3601g. Table 2 also shows the relation between zBW category and birth weight. Macrosomic participants (2.7% of all) had mean birth weight 4739g (SD 292; range 3870–7270).

**Table 2:** Distribution of independent variables and their relations to birth weight and perinatal mortality: 1 925 929 infants born in Norway 1967–2011.

Category	Number	%	Mean birth weight g (SD)	PNM per 1000
*All*	1 925 929	100	3601 (497)	4.2
zBW category (z-score)				
<-4	568	0.0	1314 (384)	410.2
-4 to -3	3149	0.2	2003 (262)	148.9
-3 to -2	31 797	1.7	2515 (241)	29.6
-2 to -1	241 137	12.5	2975 (226)	8.1
-1 to 0	691 106	35.9	3381 (234)	3.4
0 to +1	662 727	34.4	3798 (239)	2.2
+1 to +2	243 417	12.6	4233 (239)	2.0
+2 to +3	45 704	2.4	4681 (239)	3.7
+3 to +4	5707	0.3	5110 (245)	8.1
>+4	617	0.0	5612 (314)	29.2
*Covariates based on family characteristics*				
Mean sibship zBW (zBW of all mother’s births)				
>2 SD lower than mean	10 716	0.6	2517 (448)	26.6
0–2 SD lower than mean	953 558	49.5	3308 (371)	5.1
0–2 SD higher than mean	939 050	48.8	3886 (388)	3.0
>2 SD higher than mean	22 605	1.2	4672 (410)	3.7
Deviance from mean sibling zBW				
≥1 SD lower	259 652	13.5	3229 (477)	12.9
0.5 to 1 SD lower	295 940	15.4	3413 (419)	4.6
<0.5 SD difference	824 840	42.8	3590 (423)	2.6
0.5 to 1 SD higher	291 358	15.1	3792 (435)	2.1
≥1 SD higher	254 139	13.2	4016 (487)	2.5
Paternity				
Same father	1 658 571	86.1	3606 (495)	4.4
Not same father	264 302	13.7	3574 (505)	3.3
Uncertain	3056	0.2	3472 (491)	6.2
Sibship size (mother’s number of infants)				
2	925 022	48.0	3581 (490)	1.8
3	697 882	36.2	3619 (497)	5.0
4	217 727	11.3	3620 (510)	9.3
5 or more	85 298	4.4	3619 (521)	10.5
*Potential confounders*, *individual*				
Year of birth				
1967–1998	1 371 445	71.2	3584 (497)	5.0
1999–2011	554 484	28.8	3643 (494)	2.3
Sex				
Female	939 746	48.8	3532 (481)	4.1
Male	986 183	51.2	3666 (502)	4.3
Birth order				
First	710 188	36.9	3511 (480)	5.2
Second	741 014	38.5	3641 (489)	3.1
Third	335 146	17.4	3675 (506)	3.9
Fourth	95 265	5.0	3668 (520)	5.4
Fifth or higher	44 316	2.3	3675 (540)	7.5
Gestational weeks				
37–38	276 742	14.4	3286 (487)	8.9
39–41	1 398 350	72.6	3634 (472)	3.1
≥42	250 837	13.0	3765 (500)	5.0
Birth defect				
No	1 870 106	97.1	3602 (495)	3.5
Yes	55 823	2.9	3554 (553)	26.7
Preeclampsia^a^				
No	1 851 214	96.1	3605 (492)	4.1
Yes	74 715	3.9	3496 (596)	8.2
Maternal age at delivery (years)				
-19	101 107	5.2	3476 (480)	5.8
20–24	498 092	25.9	3539 (485)	4.6
25–29	673 111	34.9	3609 (491)	3.9
30–34	461 002	23.9	3657 (499)	3.7
35–39	166 821	8.7	3667 (514)	4.7
40+	25 796	1.3	3650 (530)	5.7
Maternal marital status				
Married/cohabitant	1 763 809	91.6	3611 (496)	4.1
Other	162 120	8.4	3493 (496)	5.4
Maternal diabetes				
No	1 912 085	99.3	3600 (496)	4.2
Yes	13 844	0.7	3799 (567)	6.6
Maternal chronic disease^b^				
No	1 802 879	93.6	3600 (495)	4.3
Yes	123 050	6.4	3616 (519)	3.7
Maternal smoking (1999–2011)				
Never	380 616	68.6	3668 (490)	2.1
Former	8380	1.5	3679 (485)	2.1
Current	77 179	13.9	3546 (503)	2.8
Did not respond	88 309	15.9	3613 (495)	2.9
Birth/placenta weight ratio (1999–2011)				
Lowest quartile (<4.832)	133 960	24.2	3611 (517)	2.6
Second (4.832 to 5.397)	133 908	24.2	3649 (493)	1.6
Third (5.398 to 6.106)	133 821	24.1	3657 (486)	2.0
Highest quartile (>6.016)	133 571	24.1	3654 (479)	3.0
Missing placenta weight	19 224	3.5	3639 (490)	3.6
Maternal prepregnancy BMI^c^				
Underweight (<18.5)	2048	3.3	3381 (441)	3.4
Normal weight (18.5–24.9)	37 512	60.3	3596 (463)	1.7
Overweight (25–29.9)	14 836	23.8	3708 (485)	2.5
Obese (≥30)	7813	12.6	3767 (517)	3.7
*Mediators*				
Obstetrical procedure^d^				
No	1 565 043	81.5	3598 (483)	1.0
Yes	355 211	18.5	3623 (543)	2.4
Dystocia^e^				
No	1 654 988	86.2	3584 (490)	1.3
Yes	265 266	13.8	3716 (507)	1.2
Birth injury^f^				
No	1 916 501	99.8	3602 (494)	1.3
Yes	3753	0.2	4124 (583)	4.0

BMI: Body Mass Index; PNM: perinatal mortality; SD: standard deviation; zBW: z-score birth weight, standardized for year of birth, sex, birth order, gestational week, and plurality.

^a^ Including any registration of eclampsia, HELLP syndrome, or hypertension.

^b^ Any registration of asthma, urinary tract disease, chronic renal disease, hypertension, rheumatoid arthritis, cardiac disease, epilepsy, thyroid disease, or pregestational diabetes mellitus.

^c^ In a 40% sample of births, 2006–2011.

^d^ Restricted to 1 920 254 live born infants; forceps, vacuum, caesarean delivery, manual extraction of placenta, curettage, or episiotomy.

^e^ Restricted to 1 920 254 live born infants; delayed childbirth due to mechanical disproportion, augmented labor, or slow progress.

^f^ Restricted to 1 920 254 live born infants; intracranial bleeding, clavicle fracture, plexus injury.

We recorded 8115 perinatal deaths (PNM 4.2 per 1000); 5775 (70%) being stillbirths and 2440 early neonatal deaths. Only 751 of stillbirths occurred during delivery, 3731 before delivery, and the remaining 1193 had unspecified timing. The relation between zBW category and PNM in Table 2 demonstrates the inverted J-pattern. A closer examination of the nadir showed a minimum PNM of 2.0 in a zBW plateau between +0.7 and +1.7. The Pearson correlation coefficient between zBW and mean sibling zBW was 0.51. Table 2 also provides relations between birth weight and PNM across covariate categories. Mostly, patterns were in accord with expectation.

### Associations between perinatal death and zBW

**Fig 3:**
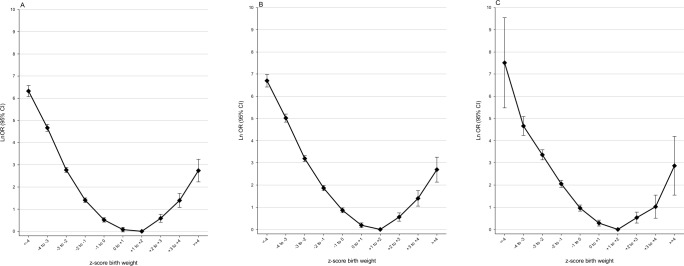
Associations between perinatal death and zBW in 3 different analytic models. CI: confidence interval; OR: odds ratio; zBW: z-score birth weight, standardized for year of birth, sex, birth order, gestational week, and plurality. (A) Model 1, crude (B) Model 2, includes potential family and individual level confounders (C) Model 4, fixed-effects model including 23 546 participants in 7686 families, discordant with respect to perinatal death. Model details provided in Table 1.

**Fig 4:**
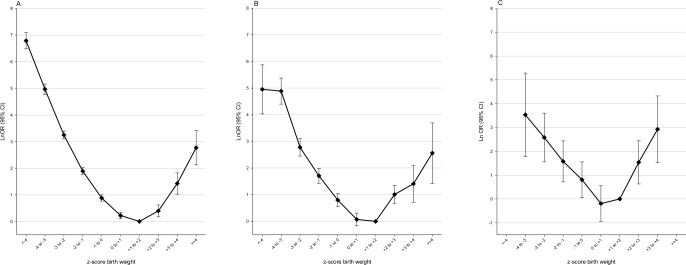
Associations between perinatal death and zBW in selected population subsets. BMI: Body Mass Index; CI: confidence interval; OR: odds ratio; zBW: z-score birth weight, standardized for year of birth, sex, birth order, gestational week, and plurality. (A) Births 1967–1998, analysis in a model including potential confounders in Model 2 (B) Births 1999–2011, analysis in a model including maternal smoking and birth/placenta weight ratio in addition to potential confounders in Model 2 (C) Births 2006–2011, analysis in a 40% sample of births, in a model including maternal prepregnancy BMI in addition to potential confounders in Model 2. See Table 1 foot-note for details.

Fig 3 shows associations between zBW and PNM in different analytical models. The inverted J-pattern in the crude Model 1 (Fig 3A) remained almost unchanged in the confounding-adjusted Model 2 (Fig 3B), and the fixed effects Model 4 (Fig 3C). The same pattern was evident for 1967–1998 births (Fig 4A). The pattern for 1999–2011 births (Fig 4B) was slightly different, with a smaller coefficient for the extreme low zBW category, and a larger coefficient for the zBW category +2 to +3, which constituted nearly 90% of all macrosomic infants (Table 2). The analysis in the maternal BMI subset showed an inverted J-pattern, but estimates had wide CIs (Fig 4C). Each potential confounder (including factors in the 1999–2011 and the BMI subset) had only marginal influence on the zBW–PNM associations. Fig 4A–4C have a pattern where the macrosomic hook is preserved in earlier and more recent births whereas the microsomic long arm of the mortality curve tends to decrease over time.

The synergistic pattern of maternal diabetes and zBW on PNM is shown in the left-hand columns of Table 3. For simplicity, only results for birth weight categories above mean (zBW>0) are included. The PNM differences were 3.9 (zBW +2 to +3), 8.2 (zBW +3 to +4), and 15.8 (zBW >+4) stronger in the diabetic compared to the non-diabetic subgroup. Collapsing the three macrosomic categories yielded a crude PNM excess of 6.2 (95% CI +0.6 to +11.8) in the diabetic compared to the non-diabetic subgroup. The random-effects regression analysis shows somewhat higher LnOR estimates in the diabetic categories, but the macrosomic hook prevailed in both non-diabetic and diabetic pregnancies.

**Table 3:** Associations between perinatal death and an interaction term of zBW and observed or hypothetical maternal diabetes: 958 172 infants with higher than mean zBW, Norway 1967–2011.

Diabetes status and zBW category (SDs from mean)	Observed maternal diabetes affecting 1% of births					Hypothetical maternal diabetes affecting 6% of births			
	Number	PNM	Difference^a^	LnOR^b^	95% CI	Number	PNM	LnOR^b^	95% CI
Not diabetes	948 685	2.2				901 250	2.0		
zBW 0 to +1	658 279	2.2	0.2	0.1	0.0 to 0.2	636 039	2.1	0.2	0.1 to 0.3
zBW +1 to +2	240 257	2.0	0	0	Reference	224 457	1.8	0	Reference
zBW +2 to +3	44 282	3.5	1.5	0.6	0.4 to 0.7	37 172	2.2	0.2	–0.1 to 0.4
zBW +3 to +4	5316	7.3	5.3	1.4	1.0 to 1.8	3361	1.2	–0.4	–1.4 to 0.6
zBW >+4	551	27.2	25.2	2.7	2.1 to 3.3	221	0.0	-	-
Diabetes	9487	6.1				56 922	6.1		
zBW 0 to +1	4448	4.5	0.1	1.0	0.5 to 1.5	26 688	4.5	1.0	0.8 to 1.2
zBW +1 to +2	3160	4.4	0	0.9	0.4 to 1.5	18 960	4.4	0.9	0.7 to 1.1
zBW +2 to +3	1422	9.8	5.4	1.7	1.1 to 2.3	8532	9.8	1.7	1.5 to 2.0
zBW +3 to +4	391	17.9	13.5	2.4	1.6 to 3.3	2346	17.9	2.4	2.0 to 2.8
zBW >+4	66	45.5	41.0	3.7	2.4 to 5.0	396	45.5	3.4	2.8 to 4.0

CI: confidence interval; PNM: perinatal mortality per 1000 births; OR: odds ratio; SD: standard deviation; zBW: z-score birth weight, standardized for year of birth, sex, birth order, gestational week, and plurality.

^a^ PNM difference between category and reference category (zBW +1 to +2).

^b^ Random-effects models including variables as in Model 2, with the exception that the 10-category interaction term is substituted for zBW and maternal diabetes (see Table 1 foot-note for details).

### Associations between perinatal death and macrosomia

Associations for macrosomia were estimated in the confounder-adjusted Model 2 in which zBW categories >+2 were collapsed (Table 4). Macrosomia was associated with PNM in the total population with an OR = 2.0. The estimates for girls and boys were quite similar whereas a stronger OR estimate for recent (1999–2011) than for early (1967–1998) births indicated heterogeneity. The difference between the two coefficients (1.069 *vs* 0.563) was significant (z = 2.58; p = 0.010) and the ratio of the two ORs (2.9/1.8) was 1.7 (95% CI 1.1 to 2.4). The explanation was a different secular trend in PNM across zBW categories: PNM for microsomic infants and normosomic infants was strongly reduced between the two periods, while PNM among macrosomic infants remained the same (4.5 in 1967–1998 and 4.4 in 1999–2011). This also meant that the PNM fraction related to macrosomia rose from 0.023 in 1967–1998 (160/6815) to 0.055 in 1999–2011 (71/1300).

**Table 4:** Associations between perinatal death and macrosomia (zBW >+2) analysed in selected population subsets and for different outcome categories: Norway 1967–2011.

Outcome and subset	Deaths per 1000	Ln OR^a^	OR^a^ (95% CI)
Perinatal death			
All	4.2	0.692	2.0 (1.7 to 2.4)
Girls	4.1	0.669	2.0 (1.5 to 2.5)
Boys	4.3	0.712	2.2 (1.6 to 2.5)
Births 1967–1998	5.0	0.563	1.8 (1.4 to 2.1)
Births 1999–2011	2.3	1.069	2.9 (2.1 to 4.0)
zBW >1 SD higher than sibling mean	2.5	0.771	2.2 (1.7 to 2.7)
zBW <1 SD higher than sibling mean	4.5	0.402	1.5 (1.1 to 2.0)
Stillbirth	2.9	0.835	2.3 (1.9 to 2.8)
Early neonatal death^b^	1.4	0.334	1.4 (1.0 to 2.0)

CI: confidence interval; SD: standard deviation; OR: odds ratio; zBW: z-score birth weight, standardized for year of birth, sex, birth order, gestational week, and plurality.

^a^ Model 2 including potential confounders, see Table 1 for details. Reference: zBW +1 to +2.

^b^ Analysis restricted to 1 920 254 live born infants in 830 124 sibships.

The crude Model 1 coefficient for macrosomia was 0.786 (OR 2.2; 95% CI 1.9 to 2.6). The attenuation from Model 1 and the confounder-adjusted Model 2 (Table 4) was 12%.

Table 4 shows that macrosomia was more strongly associated with stillbirth than with early neonatal death. We assessed the mediating strength of obstetrical procedure, dystocia, and birth injury by comparing the early neonatal death coefficient in the confounder-adjusted Model 2 (0.334; Table 4) with the corresponding result in a confounder- and mediator-adjusted model (0.270). The attenuation (100 (1–0.270/0.334)) was 19%.

We also examined the macrosomia–PNM association in the fixed-effects analysis among the 23 546 infants in the 7686 discordant families (Model 4 in Table 1). In order to estimate the impact of shared confounders, we compared the fixed-effect Model 4 with an unconditional logistic regression analysis for the same 23 546 infants in a model including only individual-level confounders. Model 4 yielded a macrosomia coefficient 0.630 (OR 1.9; 95% CI 1.5 to 2.4), which was 15% lower than the unconditional result (coefficient 0.739; OR 2.1; 95% CI 1.7 to 2.6).

In essence, these analyses provided estimates of 12% attenuation by measured confounders, 15% by shared confounders, and 19% by mediators (early neonatal death).

### The modifying role of sibling zBW on associations between perinatal death and zBW

**Fig 5:**
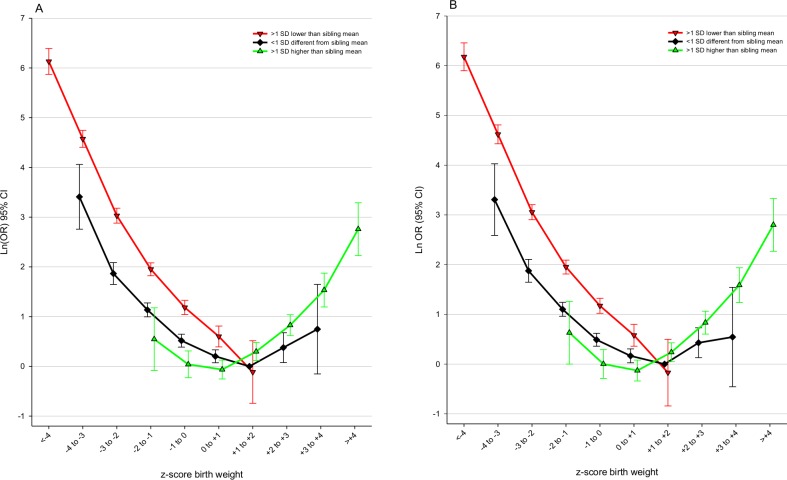
Associations between perinatal death and zBW, by deviance between own zBW and mean sibling zBW. CI: confidence interval; OR: odds ratio; SD: standard deviation; zBW: z-score birth weight, standardized for year of birth, sex, birth order, gestational week, and plurality. (A) Siblings in maternal families (B) Siblings with the same mother and father. The models include variables as in Model 2, with the exception that a 30-category interaction term is substituted for zBW and mean sibship zBW. Reference: participant zBW +1 to +2 and <1SD different from mean sibling zBW. See Table 1 foot-note for details.

Fig 5A shows that both positive and negative difference between own zBW and mean sibling zBW influenced the associations between zBW and PNM. The inverted J pattern prevailed among participants with zBW <1 SD different from siblings (black). The subset with zBW >1 SD lower than their siblings (red) had a steeper long arm and an absent hook. Participants with zBW >1 SD higher than their siblings (green) showed a different pattern with increasingly stronger coefficients for the four heaviest zBW categories, and lower coefficients than the two other subsets for low (<0) zBW categories. This yielded a pattern where PNM was higher for microsomic and normosomic infants *smaller* than their siblings, and for macrosomic infants *larger* than their siblings. The same analysis restricted to sibships with the same father (Fig 5B) showed almost identical coefficients as for all participants. Confidence intervals were however wide, in particular in the macrosomic region.

Table 4 shows that the macrosomia–PNM association was stronger (OR 2.2) for the subset where own zBW was higher than sibling mean zBW, than for those with similar or lower own zBW (OR 1.5). This is in accord with Fig 5A. We tested this difference and found a borderline significance (z = 1.949; p = 0.051; ratio of ORs 1.4; 95% CI 1.0 to 2.1). A similar analysis restricted to sibships with the same father showed a slightly stronger difference (z = 2.112; p = 0.035).

In a clinical setting, the influence of sibling zBW would have to rely on elder siblings. We examined this in 652 964 pairs of first and second born. Among the 15 370 macrosomic second born infants, 46 were perinatal deaths (PNM 3.0). PNM was 2.5 times higher for those with zBW >1 SD higher than the elder sibling (PNM 4.0) than for those with similar (<1 SD different) zBW (PNM 1.6).

The PAF estimates were based on the observed PNM_O_, and on the hypothetical PNM_H_ under the assumption that all infants had the same mortality risk as those with small (<0.5 SD) difference from sibling mean zBW. PNM_O_ and PNM_H_ were 4.2 and 3.0, respectively (Table 5). This yielded a PAF estimate of 0.29 (95% CI 0.26 to 0.32). PAFs were highest for microsomic infants, but had also considerable impact for normosomic and macrosomic infants. The PAF for infants born in 1999–2011 was 0.28 (95% CI 0.21 to 0.34).

**Table 5:** Population fraction of perinatal deaths attributed to deviance from mean sibling birth weight, according to zBW category and selected population subsets: Norway 1967–2011.

Category	% of total	Hypothetical/observed PNM^a^	PAF^b^	95% CI
All	100	3.0/4.2	0.29	0.26 to 0.32
Microsomic (zBW <–2)	1.9	14.8/46.4	0.68	0.57 to 0.77
Normosomic (zBW –2 to +2)	95.4	2.6/3.4	0.23	0.21 to 0.26
Macrosomic (zBW >+2)	2.7	2.7/4.4	0.39	0.07 to 0.60
Infants born 1967–1998	71.2	3.5/5.0	0.29	0.26 to 0.31
Infants born 1999–2011	28.8	1.7/2.3	0.28	0.21 to 0.34
Infants in sibships with the same father	86.1	3.1/4.4	0.29	0.26 to 0.32
Infants in sibships with more than one father	13.7	2.4/3.3	0.28	0.20 to 0.36
Infants with >2 siblings^c^	32.0	6.6/9.9	0.34	0.29 to 0.38
Infants with 1 or 2 siblings	68.0	1.5/2.0	0.27	0.21 to 0.32

PAF: population attributable fraction; PNM: perinatal mortality per 1000 births; zBW: z-score birth weight, standardized for year of birth, sex, birth order, gestational week, and plurality.

^a^ Applying Stata’s *punaf* command and based on a logistic regression model including zBW (10 categories), deviance from mean sibling zBW (5 categories) birth defect, preeclampsia, maternal age, maternal marital status, maternal diabetes, and maternal chronic disease.

^b^ Hypothetical PNM: PNM under the hypothetical scenario that all had the same PNM as the category of infants with similar z-score birth weight (<0.5 different) as their siblings.

^c^ Maternal diabetes omitted from model due to non-convergence.

### Sensitivity analyses

**Fig 6:**
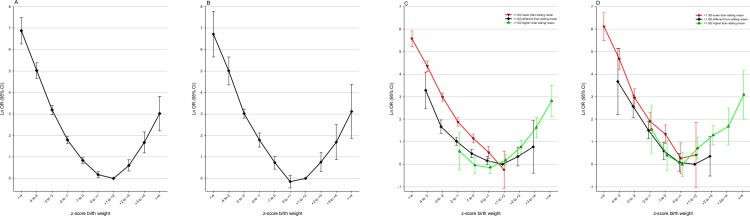
Sensitivity analysis comparing associations between perinatal death and zBW in first-born and last-born infants. CI: confidence interval; OR: odds ratio; SD: standard deviation; zBW: z-score birth weight, standardized for year of birth, sex, birth order, gestational week, and plurality. (A) First-born infants. The model includes potential confounders in Model 2 (B) Last-born infants. The model includes potential confounders in Model 2 (C) First-born infants. The model includes variables as in Model 2, with the exception that a 30-category interaction term is substituted for zBW and mean sibship zBW. Reference: participant zBW +1 to +2 and <1SD different from mean sibling zBW (D) Last-born infants The model includes variables as in Model 2, with the exception that a 30-category interaction term is substituted for zBW and mean sibship zBW. Reference: participant zBW +1 to +2 and <1SD different from mean sibling zBW. See Table 1 foot-note for details.

We identified 782 434 infants who were the mother’s first-born and 784 517 who were the mother’s last-born between 1967 and 2011. The PNM patterns according to zBW category were similar in both groups (Fig 6A, Fig 6B). We found similar patterns for first-born and last-born for PNM in association with the 30-level interaction variable (Fig 6C, Fig 6D).

We assumed that mean sibling zBW was a proxy for optimum birth weight for each participant. In analyses of subset groups expected to have different quality of this proxy, we found only minute differences in PAF estimates (Table 5). Subsets assumed to have better proxy quality (infants in sibships with the same father; infants with >2 siblings) had both slightly higher PAFs than their counterparts with assumed poorer quality.

The sensitivity analysis with the hypothetical diabetes variable showed that increasing prevalences led to increasing depletion of perinatal deaths in the non-diabetic macrosomic strata. Assuming a scenario with a sixfold increased prevalence (from the observed 1% to 6%) had as result that the majority of macrosomic deaths changed from observed non-diabetic to hypothetic diabetic category (Table 3, right-hand columns). The random-effects regression shows a macrosomic mortality hook confined to hypothetical diabetic categories, and an absent hook in the non-diabetic subgroup.

## Discussion

The study covers the majority of term and post-term born singletons in Norway during 45 years. PNM showed a clear inverted J-pattern in association with zBW. This pattern prevailed in all analyses including measured individual-level confounders, measured and unmeasured family-level (shared) confounders, and mediators. Maternal diabetes and macrosomia had an interaction (synergistic) effect on PNM. All covariates had only moderate impact on the inverted J shape and the PNM association with macrosomia. Contrary to the study hypothesis, shared confounders, as evaluated in fixed-effects analysis, did not explain the hook of the inverted J. By contrast, deviance from mean sibling zBW modified the zBW–PNM relation: the relation between macrosomia and mortality was stronger if the infant was larger than their siblings compared with the magnitude of the relation if the infant was a similar size as siblings. Similarly, the relation between microsomia and mortality was stronger if the infant was smaller than their siblings compared with the magnitude of the relation if the infant was a similar size as the siblings. This is the first study to demonstrate that the modification of PNM risk by difference in size between siblings is a general pattern that covers the entire birth weight range. The study is also the first ever to provide a quantitative estimate of the influence of deviance between own and sibling birth weight on PNM, indicating that family (sibling birth weight) is associated with a substantial portion of the variance of the birth weight–mortality relation.

### Strengths and limitations

In this large population-based study, we linked siblings in consecutive birth records in a procedure independent of maternal recall. Birth records included data on a number of relevant covariates. MBRN registration includes all infants born in Norway [47].

The participants constituted 92% of singleton term or post-term births to mothers with more than one birth in the study period. Exclusions were mainly due to missing gestational age data.

Model misspecification could be a problem in this observational study. We governed the analyses according to the causal diagram (Fig 2). Previous studies have usually considered birth weights of elder siblings [39–42]. We trusted a model in which both elder and younger siblings counted alike. The comparison of first-born and last-born infants suggests that our assumption holds. The assumed mediators constituted a specific modeling problem. Delivery events (obstetric procedures, dystocia, birth injuries) are well known correlates with birth weight and perinatal death [10–12]. A causal sequence starting with macrosomia followed by riskful delivery events and subsequent death would truly be an example of mediation as illustrated in Fig 2. However, for late fetal deaths the sequence could be different, e.g., that the decision of an obstetric procedure would follow from the fact that the macrosomic infant was dead. Confounders of the birth weight–PNM association could also independently initiate obstetric procedures. Preeclampsia could be such a factor, influencing not only birth weight and survival, but also the decision to undertake obstetric procedures. In this case, it would be difficult to disentangle the causal sequence and whether death was truly related to birth weight at all. In both instances, the conditioning on assumed mediators could generate collider (selection) bias [52]. In the end, we decided to restrict the mediation analysis to three selected mediators and early neonatal death because the majority of perinatal deaths were stillbirths before delivery. Nevertheless, caution is particularly warranted in the assessment of mediation effects in Model 3.

Information bias could pose a problem. We consider data on zBW and perinatal death to be reliable, rendering the crude zBW–PNM association reasonably valid. Associations with macrosomia could be underestimated because this is a heterogeneous entity with a complex etiology [10]. Quality problems of the sibship variables could underestimate their impact on the zBW–PNM associations. We considered mean sibship zBW to be an indicator, most likely an imperfect proxy, of the optimum birth size of the family. This was however not a problem in the fixed effects analysis where mean sibship zBW was invariant.

We were able to account for measured confounders and unmeasured shared confounders, but not for confounding by unmeasured individual factors. This could pose an important threat to valid inferences. We lacked data on potentially important pregnancy-specific factors. Examples are placenta function and metabolic characteristics that could have profound effects on birth weight and survival, and thereby have major influence on the results. We consider incomplete data on BMI and particularly maternal diabetes to be a limitation. The prevalence of diabetes in the MBRN barely reached 2% in the most recent years. This is in contrast to a many-fold higher occurrence of reported gestational diabetes in more recent studies [18]. The prevalence of gestational diabetes according to WHO criteria was 10.9% among ethnic Norwegians and other Western Europeans living in Norway in 2008–2010 [56]. The low numbers in the MBRN is probably due to incomplete use of diagnostic procedures [57]. The sensitivity analysis in Table 3 suggests that incomplete registration of conditions related to poor glycemic control could be particularly important in explaining the macrosomic mortality hook if interaction was present.

Several covariates in this register-based study were crude approximations (e.g., birth defects), and we would expect that adjustments in the multivariable models for this reason would be incomplete. Incomplete recording of some covariates could add to this problem. Except for maternal marital status and sibship size, we had no indicators of parental socioeconomic position. This probably poses a minor problem since socioeconomic status seems to have little influence on the relation between birth weight and mortality in countries such as Norway [43]. We were able to account for unmeasured shared factors in the fixed-effects analysis, but error in individual confounders can pose a larger problem in sibling analysis than in individual analysis [58].

Lack of power constituted a problem in small zBW strata, particularly for the macrosomic categories where PNM risk was lower than in the microsomic area. The same applies to some of the subset analyses and to the fixed-effects analysis because <1% of families were discordant with respect to perinatal deaths.

When assessing the generalizability of the results, one should keep in mind that the study was restricted to singleton and term or post-term births, and that it related to a relatively homogenous and low-risk population in a developed country. The study covered an extended period and results could therefore be outdated. The results for births between 1999 and 2011 suggest however that the findings are still relevant.

### Comparison with other studies and inferences

The inverse J-pattern of the mortality curve is soundly documented [1–8]. The hook of the curve is in accord with excess perinatal mortality rates in macrosomic births [10–12,14,23,24]. However, the choice of cut-off weight for macrosomia is important [10] and can explain the lack of association for weights ≥4 kilos [13]. Our results are in accord with a study by Bukowski et al. who found no stillbirth association for birth weight for gestational age at the 90^th^-95^th^ percentile but a strong association for >95^th^ percentile weights ([24], Table 3).

Evolutionary biologists interpret the inverted J-pattern on the population level a result of natural [1] or stabilizing [2] selection. One plausible explanation of our results could be that this acts not only on the population level, but also on the family level. Infants who deviate not only from a population mean, but also from the family mean, have higher risk of mortality. Similar to our findings, sibling studies addressing birth weight and survival [39–43] have shown that infants who are small relative to their siblings are at excess risk of perinatal or neonatal death. Results of family studies using maternal rather than sibling birth weight [44] point in the same direction. Mostly, these family-design studies have not addressed mortality in infants outweighing their relatives. While large, these studies have not had sufficient numbers to estimate the mortality pattern of macrosomic infants [39–44]. In some studies, macrosomic infants have not been included as own categories [40]. Pedersen et al. [41] reported a slightly higher neonatal mortality in infants with a birth weight higher than their elder sibling but had no higher birth weight category than ≥4000g.

The results suggest that factors causing deviance from optimum birth weight in the individual birth are pregnancy-specific and not shared by siblings. Numerous studies show that maternal pregestational and gestational diabetes mellitus have impact on macrosomia and perinatal mortality [16–18]. This fact combined with the interaction between maternal diabetes and macrosomia on mortality, and the sensitivity analysis in Table 3 could open for a speculative but plausible interpretation. The “hypothetical diabetes” variable in Table 3 could represent characteristics related to poor glycemic control that were of moderate prevalence and associated with macrosomia and perinatal death. Birth weight-dependent heterogeneity in mortality could then result in a scenario where a major part of the macrosomic mortality hook was restricted to the subset with these metabolic characteristics. This would be in accord with an explanation where the hook would be the result of interaction [8] rather than confounding without interaction [6]. Birth weight would not necessarily be a mediator in a causal path between the metabolic characteristic and perinatal death but could rather be an indicator of inadequate management of the metabolic characteristic in pregnancy. We are not aware of studies addressing such interaction, but this interpretation could be in concert with clinical trials of mild gestational diabetes where treatment (dietary advice, blood glucose monitoring, insulin) effected in lower macrosomia prevalence [59,60] and perinatal mortality [59] or complications that could result in perinatal death [60].

### Clinical and public health aspects

Our results underline the importance of obstetricians taking size at birth in the family into account when assessing mortality risk. Apart from obstetrical trauma, perinatal death among macrosomic infants could be heterogeneous and more of a problem in pregnancies complicated by poor glycemic control. If so, management in pregnancy according to recent guidelines [61] could prove effective in reducing perinatal mortality in macrosomic infants.

Macrosomia affects only a small proportion of all births, and only 3% of all perinatal deaths affected this weight category in the present study. Its public health impact is however increasing because macrosomia worldwide has become more common [10]. Furthermore, the relative importance of PNM in macrosomic infants has increased in Norway because the strong secular decrease in mortality has primarily benefitted microsomic and normosomic infants.

## Conclusions and future research

This sibling-designed study shows that family factors have large impact on the relation between birth weight and survival. Deviation of birth weight from sibling birth weight in either direction is associated with excess PNM. In our study, the excess among macrosomic infants was not confounded by shared family factors as hypothesized, but was evident among those who outweighed their siblings. The main explanation of the hook of the inverted J is probably found among unknown pregnancy-specific factors. Future studies should identify and include more complete and refined data on such factors, metabolic and others. Causal paths including macrosomia and perinatal mortality are intricate, and models that are more refined can probably be achieved by applying causal analytical methods [62].
